# In Vitro Antibacterial Effectiveness of a Naturopathic Oral Care Product on Oral Pathogens

**DOI:** 10.3290/j.ohpd.a44938

**Published:** 2020-07-24

**Authors:** Alina Beyer, Marly Dalton, Katharina Doll, Andreas Winkel, Nico S. Stumpp, Meike Stiesch

**Affiliations:** a Dentist, Department of Prosthetic Dentistry and Biomedical Materials Science, Hannover Medical School, Hannover, Germany. Performed the experiments in partial fulfillment of requirements for a Dr. med. dent. degree, investigation, data curation, formal analysis, visualisation, wrote the manuscript.; b Researcher, Department of Prosthetic Dentistry and Biomedical Materials Science, Hannover Medical School, Hannover, Germany. Methodology, investigation, data curation, reviewed and edited the manuscript.; c Researcher, Department of Prosthetic Dentistry and Biomedical Materials Science, Hannover Medical School, Hannover, Germany. Formal analysis, validation, visualisation, reviewed and edited the manuscript.; d Researcher, Department of Prosthetic Dentistry and Biomedical Materials Science, Hannover Medical School, Hannover, Germany. Formal analysis, supervision, visualisation, reviewed and edited the manuscript.; e Researcher, Department of Prosthetic Dentistry and Biomedical Materials Science, Hannover Medical School, Hannover, Germany. Conceptualization, methodology, formal analysis, supervision, validation, visualisation, review and editing the manuscript.; f Professor and Head of Department of Prosthetic Dentistry and Biomedical Materials Science, Hannover Medical School, Hannover, Germany. Project administration, resources, funding acquisition, conceptualisation, methodology, formal analysis, supervision, review and editing the manuscript.

**Keywords:** essential oils, medicinal plant extracts, minimum inhibitory concentration (MIC), naturopathic oral care product, periodontal pathogenic or halitosis-associated bacteria

## Abstract

**Purpose:**

Currently, the prevention of periodontal diseases focuses on mechanical removal of pathogenic biofilms combined with oral antiseptics as supportive chemical antibacterial control. Due to the risk of resistance development and side effects of existing antiseptics, the interest in alternative medicine with naturopathic treatment modalities is growing in dentistry. In the present study, the antibacterial effect of the naturopathic oral care product Repha OS and some of its derivatives, based on medicinal plant extracts and essential oils, with a specific focus on added sweeteners, was investigated on periodontal pathogenic and halitosis-associated bacteria.

**Materials and Methods:**

The antibacterial efficacy was investigated by agar dilution assay. The minimum inhibitory concentration (MIC) for the bacterial species *Aggregatibacter actinomycetemcomitans, Fusobacterium nucleatum, Porphyromonas gingivalis, Prevotella intermedia* and *Solobacterium moorei* was determined.

**Results:**

A concentration-dependent antibacterial effect on oral bacterial species by Repha OS and its derivatives was demonstrated. For the original product, the maximum MIC was 10% of the calculated test solution concentration in agar for all examined bacterial species. The removal of essential oils reduced the antibacterial efficacy, whereas the displacement or replacement of sweeteners had almost no effect.

**Conclusion:**

In addition to other individual effects of the ingredients, the results of this study show that an antibacterial effect of the naturopathic oral care product on the tested oral bacterial species was achieved in vitro. In vivo, the combination of this antibacterial effect with other properties of the various ingredients may be interesting for a holistic approach in preventive dentistry.

The resident flora of the human oral cavity shows great bacterial diversity, with more than 700 different species.^49, 69^ These are usually in ecological balance with the host organism. However, under certain conditions, the bacterial composition can shift and promote the integration of pathogenic bacteria, subsequent opportunistic infection and periodontal disease.^35^

Periodontal disease includes inflammatory disorders of all periodontal tissues: gingiva, periodontal ligament, cementum and alveolar bone.^45^ It can manifest in a milder form as gingivitis, a reversible inflammation of the gums, or in a severe form as periodontitis, which progressively leads to an irreversible destruction of affected periodontal tissues with persistence of inflammation and bone loss.^34^ Beside the increased proportion of facultative pathogenic bacteria, the unhindered development and spread of dental plaque – highly organized bacterial agglomerates – are the main causes for periodontal disease.^5,60^ Frequent mechanical removal of these biofilms by toothbrushing, professional scaling or root planing is essential to prevent and treat any periodontal disease.^45^ Unfortunately, mechanical plaque control shows limitations in terms of accessibility^3,43^ or possible further tissue damage.^42,55,75^

Therefore, the additional use of oral antiseptics to reduce the overall bacterial load in daily oral care can be appropriate, e.g. in elderly patients with limited manual dexterity.^26^ This becomes a more important topic in an ever-aging society with an increasing proportion of older people and patients due to demographic changes. Although the current Fifth German Oral Health Study from 2014 (DMS V) shows a declining trend in serious periodontal disease among both younger adults (aged 35 to 44 years) and younger elderly (aged 65 to 74 years), the need for periodontal treatment will increase.^33^ On the one hand, this is caused by a shift of chronic oral diseases, such as periodontitis, to a higher age. On the other hand, in addition to a general preventive attitude, a trend towards tooth preservation in older patients is evident in dentistry, as the DMS V shows on the basis of a significant decline in tooth loss.^33^

Therefore, also preventive chemical antibacterial control with oral antiseptics could be a useful adjunct to mechanical biofilm removal without completely replacing it.^3^ The successful adjuvant application of antiseptic solutions, especially preparations containing chlorhexidine, has already been shown by many authors in preventive dentistry and especially periodontal therapy.^10,47,56^ Chlorhexidine may be considered the ‘gold standard’ in oral antiseptic therapy^56^ due to its broad antimicrobial spectrum^6,48,70^ and high substantivity.^29,32,41^ But there may be several local side effects depending on the duration of use and the concentration of chlorhexidine. These may include discolouration of teeth and tongue, taste alterations, mucous membrane erosion and delayed or disturbed wound healing.^20,21,56^ In addition to the side effects of conventional chemical antiseptics, a major problem in the current treatment of periodontal disease is the risk of developing resistance to antimicrobials.^45^ While the problem of a potential bacterial resistance to antibiotics has been known for years, a possible bacterial adaptation to oral antiseptics has long been underestimated. Several studies indicate a possible cross-resistance between antibiotics and antiseptics,^37,71^ such as chlorhexidine^14,50^ or triclosan,^13,53^ which may lead to multidrug resistance in dental plaque bacteria.^45^ In addition to the necessary research on such bacterial resistance mechanisms,^14^ there is also a need for alternative treatment modalities. Thus, the interest in naturopathic treatment methods is also increasing in the dental field,^63^ reflected by the large number of pertinent in vitro and in vivo studies on medicinal plant extracts.

The commercially available oral care product Repha OS (Repha; Langenhagen, Germany) contains extracts from tormentil, ratanhia and myrrh as the main components, with additives of peppermint, eucalyptus, cloves and aniseed oil, as well as limonene, linalool, levomenthol, saccharin or stevia, and 69% ethanol.^54^ Some of these medicinal plant extracts and essential oils are known to have antibacterial properties.^2,51,61,65,72^

A previous in vitro study already demonstrated the growth inhibition of several gut-, skin- and mucosa-associated bacterial species in an aerobic agar dilution assay.^4^ However, the antibacterial effect of the oral care product Repha OS on anaerobic bacterial pathogens causing oral disease has not yet been investigated. The aim of the present study was to analyse the antibacterial effect of the Repha OS oral care product for the first time on the growth of obligatory or facultative anaerobic, periodontal pathogenic and halitosis-associated bacterial species in vitro by using an agar dilution assay. The contribution to this effect by the main components – extracts of medicinal plants and essential oils – was determined. Additionally, the influence of added sweeteners, saccharin or stevia, on the antibacterial efficacy was investigated.

## Materials and Methods

### Test Organisms and Cultivation

Microorganisms were obtained from the German Collection of Microorganisms and Cell Cultures (DSMZ) of the Leibniz Institute in Braunschweig, Germany. Four gram-negative bacterial strains [*Aggregatibacter actinomycetemcomitans* (DSM 11123), *Fusobacterium nucleatum* (DSM 15643), *Porphyromonas gingivalis* (DSM 20709), *Prevotella intermedia* (DSM 20706)] and one gram-positive bacterial strain [*Solobacterium moorei* (DSM 22971)] were used.

Fastidious anaerobe agar (FAA) was selected for the cultivation of test organisms on solid medium (LAB M; Heywood, England). Before the plates were prepared, the agar solution was supplemented with 5% sterile defibrinated sheep blood (Oxoid Deutschland; Wesel, Germany). The agar plates were freshly produced for the cultivation of the pre-cultures as well as for the trial preparation and were not stored for long periods.

For pre-cultures, bacterial stock cultures were streaked on agar plates and incubated for at least 48 h (*F. nucleatum, P. gingivalis*) or 24 h (*A. actinomycetemcomitans, P. intermedia, S. moorei*) at 37°C under anaerobic conditions (80% N_2_, 10% H_2_, 10% CO_2_; anaerobic workbench, Concept 400, Baker Ruskinn; Bridgend, UK).

### Agar Dilution Assay Preparation

The investigated products were obtained either in the original packaging (Repha OS oral spray, LOT: 52003, BBD: 07/2018) or as bottles (Repha OS without saccharin, Lab code: NH0143; Repha OS without saccharin and essential oils, Lab code: NH0143; Repha OS with stevia for substitution of saccharin, Lab code: 347) from the manufacturer’s production (Repha). As all products contained 69% ethanol as solvent, a 69% ethanol solution (J. T. Baker; Schwerte, Germany) was used as control.

The antibacterial efficacy of the investigated products was examined by agar dilution assay according to the protocol described by Beckmann et al.^4^ The agar plates were prepared from FAA with sheep blood, as mentioned above, and additionally supplemented with the products in a serial dilution to final concentrations of 0.63% to 20%. To prevent ethanol evaporation, the test solutions were added to the liquid agar at a temperature of approximately 46°C. The temperature was monitored by an infrared thermometer (Fluke 62 MAX, Fluke Deutschland; Glottertal, Germany). Agar plates without additional supplements served as control growth plates.

### Bacterial Inoculation

Three separate pre-cultures were prepared per bacterial species to achieve three biological replicates. To produce inocula with equal bacterial concentrations, several single colonies were removed from each agar plate, resuspended in sterile 0.9% NaCl solution (*A. actinomycetemcomitans, P. intermedia, S. moorei*) or in Schaedler nutrient medium (Oxoid Deutschland; *P. gingivalis, F. nucleatum*) and adjusted to the adsorption of McFarland standard 0.5 (barium sulfate standard, Remel Products; Lenexa, KS, USA). The absorption was determined at 600 nm (OD 600) using a photometer (BioPhotometer D30, Eppendorf Liquid Handling; Hamburg, Germany).

**Fig 1:**
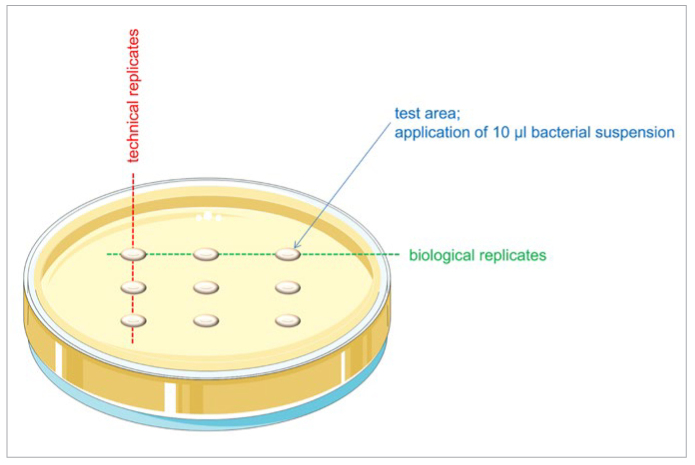
Application scheme of the bacterial suspensions.

Ten μl of the adjusted bacterial suspensions was dotted onto the supplemented agar plates. Three technical replicates of each biological replicate were applied per agar plate (Fig 1). These steps were carried out in the anaerobic workbench, where incubation also took place under anaerobic conditions (80% N_2_, 10% H_2_, 10% CO_2_) at 37°C for 48–72 h until bacterial growth was macroscopically visible on control plates.

### Trial Evaluation

After incubation, the growth events on the agar plates were documented photographically. For every concentration, the macroscopic detectability of bacterial growth was rated as yes/no. The respective test was only valid if the growth control was positive. In this case, the agent-free growth controls showed circular bacterial growth of about 0.5 cm on all test areas (Fig 1).

Using this agar dilution assay, the minimum inhibitory concentration (MIC) of the investigated products was determined for each test organism according to the selected test parameters. The MIC was defined as the dilution level at which no bacterial growth occurred macroscopically in any of the replicates. For fine gradation, all positive growth events (GE) of the next greater dilution level were additionally quantified, up to the point at which bacterial growth was observed for the last time before reaching the MIC.

## Results

The antibacterial efficacy of the Repha OS oral care product and its derivatives was analysed by agar dilution assay according to the protocol by Beckmann et al.^4^ The number of growth events on agar plates supplemented with different concentrations of the naturopathic products was counted manually. The results showed that all investigated variants of the product exhibited a clear antibacterial effect on the analysed bacterial species, but differed in concentration.

**Fig 2:**
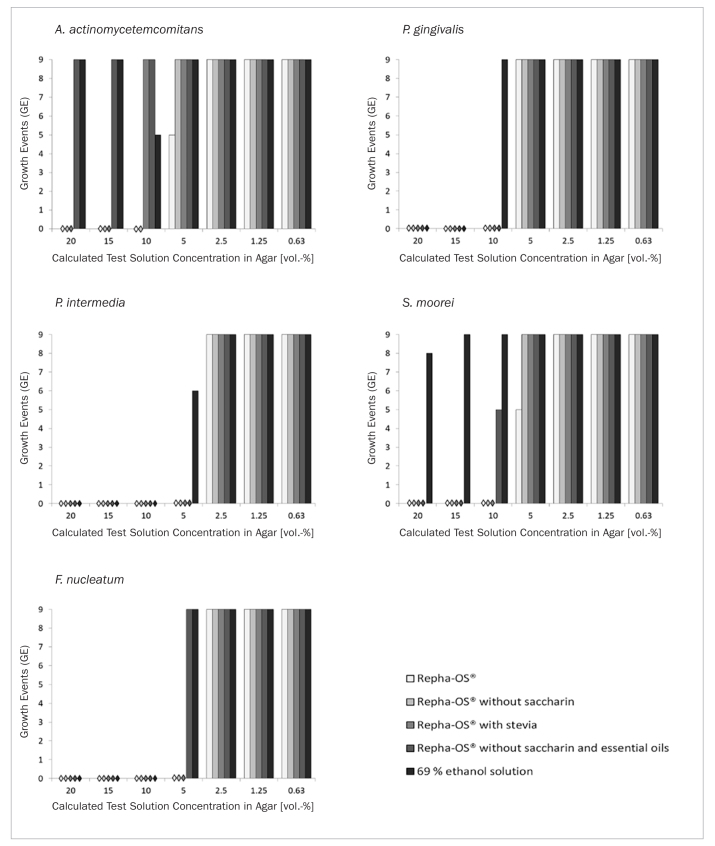
Growth events (GE) of *A. actinomycetemcomitans, P. gingivalis, P. intermedia, S. moorei* and *F. nucleatum* treated with different Repha OS products and an ethanol control solution at different concentrations. According to the experimental setup, a maximum of 9 positive GEs was possible (Fig 1).

**Fig 3:**
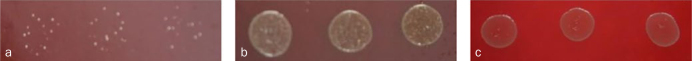
Bacterial growth of *F. nucleatum* at a calculated test solution concentration in agar of 2.5% after incubation for 72 h for Repha OS (a), Repha OS without saccharin (b) and Repha OS with stevia (c).

The detailed bacterial growth events for every combination are shown in Fig 2 for *A. actinomycetemcomitans, P. gingivalis, P. intermedia, S. moorei* and *F. nucleatum*. Additionally, as an example, Fig 3 demonstrates different bacterial growth morphologies of *F. nucleatum* at a calculated test solution concentration in agar of 2.5% after incubation for 72 h with Repha OS (A), Repha OS without saccharin (B) and Repha OS with stevia (C). The MIC determined for every combination and the mean number of GEs at the next lower concentration are listed in Table 1.

**Table 1:** MIC of test organisms treated with different Repha OS products

	*A. actinomycetemcomitans*		*P. gingivalis*		*P. intermedia*		*S. moorei*		*F. nucleatum*	
	MIC	GE	MIC	GE	MIC	GE	MIC	GE	MIC	GE
Repha OS	10%	5% (5/9)	10%	5% (9/9)	5%	2.5% (9/9)	10%	5% (5/9)	5%	2.5% (9/9)
Repha OS without saccharin	10%	5% (9/9)	10%	5% (9/9)	5%	2.5% (9/9)	10%	5% (9/9)	5%	2.5% (9/9)
Repha OS with stevia	15%	10% (9/9)	10%	5% (9/9)	5%	2.5% (9/9)	10%	5% (9/9)	5%	2.5% (9/9)
Repha OS without saccharin and essential oils	>20%	20% (9/9)	10%	5% (9/9)	5%	2.5% (9/9)	15%	10% (5/9)	10%	5% (9/9)
69% ethanol solution	>20%	20% (9/9)	15%	10% (9/9)	10%	5% (6/9)	>20%	20% (8/9)	10%	5% (9/9)

MIC is shown as calculated test solution concentration on agar plates, where no bacterial growth was detected in any of the replicates. If no inhibition of bacterial growth was observed for all concentrations tested, it was indicated as >20%. GE is the calculated test solution concentration on agar plates, where the last growth events were observed. The mean number of GEs at this concentration is shown in brackets. According to the experimental setup, a maximum of 9 positive GEs per concentration was possible (Fig 1).

For the original product (Repha OS), the maximum MIC was at 10% of the calculated test solution concentration for the bacterial species *A. actinomycetemcomitans,* *P. gingivalis* or *S. moorei*, and even lower (at 5%) in the case of *P. intermedia* or *F. nucleatum*. The displacement of saccharin caused no changes in the MICs, but increased the number of positive GEs at the next lower concentration from 5 to 9 out of 9 for the bacterial species *A. actinomycetemcomitans* and *S. moorei*.

Where stevia substitued for saccharin, the results corresponded with the MICs and GEs of the product without saccharin for *F. nucleatum, P. gingivalis, P. intermedia* or *S. moorei*. An exception in this context was only *A. actinomycetemcomitans* with a resulting MIC of 15% instead of 10%, as observed for both Repha OS as well as Repha OS without saccharin.

Most differences compared to these similarly active products were noted for Repha OS without saccharin and essential oils, which showed lower antibacterial efficacy against 3 out of 5 oral bacterial species examined. The observed decrease for *A. actinomycetemcomitans* was particularly strong, from a MIC at 10% to unaffected growth of bacteria up to 20%. *P. gingivalis* or *P. intermedia* were the only exceptions, with no differences in the reaction regarding all investigated Repha OS products. Although no differences in the GEs and therefore also not in the resulting MICs were described for *F. nucleatum* with Repha OS, Repha OS without saccharin or Repha OS with stevia, the loose growth morphology of the bacterial colonies given the original product was another notable observation (Fig 3).

As a control, respective dilutions of 69% ethanol solution were also investigated for MICs and GEs of all tested bacterial species. In comparison, these controls showed the least inhibition of bacteria. No MICs could be determined for *A. actinomycetemcomitans* or *S. moorei* with only sporadically reduced GEs at certain concentrations (Fig 2), which were not further investigated.

## Discussion

Currently, treatment and prevention of periodontal diseases focuses on regular mechanical removal of pathogenic bacteria with simultaneous use of oral antiseptics as supportive chemical bacterial control. Because existing chemical antiseptics such as chlorhexidine can have multiple side effects with long-term or highly concentrated use,^20,21,56^ and the risk of developing bacterial resistances is on the rise,^45,71^ the interest in alternative medicine, including naturopathic treatments, is growing in dentistry. To investigate whether the commercially available oral care product Repha OS, which is based on medicinal plant extracts and essential oils, shows promising characteristics as an alternative antiseptic in preventive dentistry, the antibacterial efficacy of the product as well as its main components on the growth behaviour of periodontal pathogenic and halitosis-associated bacterial species was analysed here in vitro for the first time.

In previous studies, the antibacterial effects of the single ingredients of the Repha OS oral care product have already been shown.^2,51,61,65,72^ For example, the medicinal plant extracts tormentil and ratanhia contain tannins, which impair bacterial metabolism and nutrient uptake.^57^ Essential oils, such as those obtained from myrrh, peppermint, eucalyptus, cloves and aniseed, consist of lipophilic molecules such as terpenes,^62^ which cause changes in structure and function of bacterial cell membranes.^16^

The selection of test organisms in this study was based on their clinical relevance. The gram-positive bacterial species *S. moorei* is related to the occurrence of halitosis due to its ability to produce volatile sulphur compounds.^27,28,67^ Halitosis is a common term used to describe oral malodor that is estimated to affect 30% of the adult population.^8,40,58^ The gram-negative bacterial species *A. actinomycetemcomitans, P. gingivalis, P. intermedia* and *F. nucleatum* are highly associated with the development and progression of periodontal diseases, e.g. gingivitis and periodontitis. The current DMS V documents the prevalence of periodontal diseases (measured using the CDC/AAP case definition [Centers for Disease Control and Prevention/American Academy of Periodontology]) as follows: 51.6% moderate and severe periodontitis among adults aged 35 to 44 years and 64.6% among younger elderly (aged 65 to 74 years).^33^

As the cultivation of these obligatory or facultative anaerobic bacterial species is challenging, the highly reproducible, standardised agar dilution assay^30^ described by Beckmann et al^4^ was used for an initial investigation of potential antibacterial efficacy. With this test, the MIC and differences between several derivatives, concentrations and bacterial species could be reliably determined. The MICs are comparable to those obtained for several gut-, skin- and mucosa-associated bacterial species.^4^

The main ingredients of all investigated derivatives of the oral care product Repha OS include extracts of tormentil, ratanhia and myrrh^54^ as well as 69% ethanol, which is added to improve the solubility of various ingredients. To exclude an antibacterial effect of alcohol alone, a 69% ethanol control solution was used in direct comparison to the Repha OS products. According to the results of the agar dilution assay, ethanol yielded the lowest inhibition of bacterial growth, with MICs at least one dilution step behind the compared Repha OS derivatives. In the case of *A. actinomycetemcomitans* and *S. moorei,* no MIC was reached within the analysis range of up to 20%. Therefore, an antibacterial effect solely based on the added ethanol can be excluded.

In contrast, the simplest Repha OS derivative (without saccharin and essential oils) – only containing medicinal plant extracts of tormentil, ratanhia and myrrh – already induced a definite antibacterial effect on the investigated bacterial species in lower concentrations, except on *A. actinomycetemcomitans*. Myrrh extracts are often combined with tannic substances, such as tormentil or ratanhia,^36^ and various previous studies have published the bacterium-specific antimicrobial properties of these medicinal plant extracts.^2,65^

The separate addition of essential oils to the Repha OS oral care product (without saccharin) improved the antibacterial properties of the product even more. Especially for *A. actinomycetemcomitans*, the benefit of these supplements is obvious, as an MIC was determined after no inhibition for ethanol alone or in combination with medicinal plant extracts. This potential synergistic effect of essential oils is supported by the literature, which describes bacteria-specific effects in several studies.^51,61,72^ However, in the case of *P. gingivalis* and *P. intermedia*, no further improvement was obtained, which supports the assumption that ethanol-dissolved plant extracts, included in all Repha OS test products, are already sufficient to inhibit the growth of these species to the highest degree.

Beside the antimicrobial effects in this experimental setting, some of the investigated medicinal plant extracts, essential oils and additional supplements were also described as possessing anti-inflammatory (myrrh,^68,74^ eucalyptus oil^59^), astringent (tormentil^36^), antipyretic (myrrh^68^), analgesic (eucalyptus oil^59^) or local anaesthetic (menthol/linalool^23^) properties. Therefore, the combination with other characteristics, attributed to the different ingredients of Repha OS, may achieve a holistic effect,^45^ which could be particularly beneficial in periodontology.

Since the combination and proportion of medicinal plant extracts and essential oils are accompanied by strong, characteristic aromas and tastes, which may not be pleasing to some consumers, sweeteners are also added to Repha OS to foster frequent and regular application. Non-caloric, so-called high-intensity sweeteners are of particular interest for the promotion of oral health, as they appear to be less cariogenic sucrose substitutes, making them also suitable for diabetes patients.^44^ Diabetes mellitus is of particular medical concern, as it is an example of a systemic disease in a bidirectional negative relationship with oral diseases such as periodontitis.^11,19^

One of the earliest sugar substitutes is saccharin, first produced in 1879 by Remsen and Fahlberg.^73^ It is widely used as a component of oral care products and is commercially available to patients. In the literature, a general antibacterial effect of saccharin against different bacterial species has been demonstrated by a large number of studies.^38,39,46^ However, the mechanisms of saccharin’s influence on bacteria have not yet been conclusively determined.^52^ In our study, the presence (Repha OS) or absence of saccharin (Repha OS without saccharin) caused no changes in the MICs in any of the investigated bacterial species. However, in the presence of saccharin, *A. actinomycetemcomitans* and *S. moorei* showed a decreased number of positive growth events at the next lower concentration. For *F. nucleatum,* the macroscopic evaluation of bacterial growth showed only a growth retarding effect of saccharin (Fig 3). In contrast to the study by Prashant et al,^52^ no noticeable influence of saccharin on *P. gingivalis* could be demonstrated here.

However, as there are indications that saccharin might mediate glucose intolerance,^64^ the more recent sugar alternative, stevia, which is a mixture of substances obtained from the leaves of the plant *Stevia rebaudiana,*^22^ was also analysed. Its main components include steviol glycosides, such as diterpene glycoside and rebaudioside A, whose proportions differ according to plant variety and area of cultivation. In terms of diabetes, the antihyperglycaemic and insulinotropic effects of stevia achieved in animal experiments are promising.^12,31^ Furthermore, a general in vitro antibacterial effect in association with stevia was reported.^1,24,66^ Especially its ability to inhibit oral pathogens,^22,25^ its non-cariogenic character,^9,17,44^ antiplaque effect^18^ and anti-inflammatory properties^7,15^ qualify it as an interesting candidate for oral applications. The MIC/GE values in our study demonstrated that the replacement of saccharin with stevia (Repha OS with stevia) reduced the antibacterial effect on *A. actinomycetemcomitans* by one dilution step. For *S. moorei*, the number of positive growth events at 5% increased, which also indicates a slightly weaker antibacterial effect. All other test organisms responded equally to the presence of both saccharin and stevia, which also corresponded to the absence of all sugar substitutes (Repha OS without saccharin).

Taken together, an antibacterial effect of the original Repha OS oral care product on all oral pathogens investigated was found for the first time, with a maximum MIC of 10% of the calculated test solution. This concentration is likely to be reached during oral application^4^ and Repha OS may thus be suitable for preventive bacteria control. The antibacterial efficacy is mainly related to the incorporated medicinal plant extracts and essential oils, whereas additional sweeteners have only a minor impact. Therefore, a change in the formula of the Repha OS oral care product is possible without sacrificing the desired properties.

## Conclusion

This study investigated the antibacterial effect of the commercially available naturopathic oral care product Repha OS and some of its derivatives, based on medicinal plant extracts and essential oils, with an additional focus on added sweeteners. All preparations analysed exhibited a concentration-dependent antibacterial effect on five clinically relevant, periodontal pathogenic and halitosis-associated bacterial species. For the original product, the MIC was 10% at most of the calculated test solution concentrations in agar for all examined bacterial species. The addition of saccharin or stevia showed only a minor influence, whereas the removal of essential oils clearly decreased the antibacterial efficacy.

Future studies should focus on the exact mode of action of such naturopathic oral care products, as well as their possible side effects, depending on dosage and duration of application. For in vivo treatment, the extent to which an oral care product in spray form can penetrate the gingival sulcus or plaque in a sufficient concentration during application should also be addressed, as the analysed bacterial species are predominantly located there in highly organised biofilms.

According to the mechanisms described for the single ingredients and the results of this study, an antibacterial effect of Repha OS on pathogenic oral biofilms can be expected but requires further investigation.
